# Supplementary material for: Impact of vaccination and risk factors on COVID-19 mortality amid delta wave in Libya: A single center cohort study
Source: PLoS One. 2023 Aug 4;18(8):e0289490. doi: 10.1371/journal.pone.0289490 (PMC10403102; doi:10.1371/journal.pone.0289490)
Supplement: Attachment — Submitted filename: Review-COVID-19 Study-MA.docx [file pone.0289490.s002.docx]

The paper is very well written, but I have following questions.

- What was the reason for selecting a prospective and a retrospective cohort? Please explain the difference of doing both
- How the participants were selected to be part of cohort
- How was the sample size derived?
- Do you think the sample is enough for a cohort study – this might me a limitation?
- Was any randomization done at any stage during sample selection?
- What was the process of taking consent from the participants in accessing the medical records

Thanks so much.
